# Empagliflozin Protects Against Doxorubicin Cardiotoxicity: Integrative Assessment of Cardiac Kinetics and Electrophysiology Using Machine Learning in a Rat Model

**DOI:** 10.3390/medsci14030342

**Published:** 2026-06-24

**Authors:** Iacob-Daniel Goje, Valentin Laurențiu Ordodi, Florina Maria Bojin, Greta-Ionela Goje, Alexandru Harald Bătrîn, Taddeus Paul Buica, Maria Iordache, Manuela Grijincu, Virgil Păunescu, Daniel-Florin Lighezan

**Affiliations:** 1 Department of Medical Semiology I, “Victor Babeș” University of Medicine and Pharmacy, No. 2 Eftimie Murgu Square, 300041 Timisoara, Romania; daniel.goje@umft.ro (I.-D.G.); dlighezan@umft.ro (D.-F.L.); 2 Advanced Cardiology and Hemostaseology Research Center, “Victor Babeș” University of Medicine and Pharmacy, No. 2 Eftimie Murgu Square, 300041 Timisoara, Romania; barbulescu.greta@umft.ro; 3 Department of Functional Sciences, Immuno-Physiology and Biotechnologies Center, “Victor Babeș” University of Medicine and Pharmacy, No. 2 Eftimie Murgu Square, 300041 Timisoara, Romania; florinabojin@umft.ro (F.M.B.); grijincu.manuela@umft.ro (M.G.); vpaunescu@umft.ro (V.P.); 4 OncoGen—Center for Gene and Cell Therapies in Cancer Treatment, Clinical Emergency County Hospital “Pius Brinzeu” Timisoara, No. 156, Liviu Rebreanu, 300723 Timisoara, Romania; alex.batrin.ab@gmail.com (A.H.B.); taddeus.b90@gmail.com (T.P.B.); 5 Faculty of Industrial Chemistry and Environmental Engineering, “Politehnica” University Timisoara, No. 2 Victoriei Square, 300006 Timisoara, Romania; 6 Department I of Nursing, Discipline of Clinical Skills, “Victor Babeș” University of Medicine and Pharmacy, No. 2 Eftimie Murgu Square, 300041 Timisoara, Romania; 7 Department of Internal Medicine, Discipline of Hematology, “Victor Babeș” University of Medicine and Pharmacy, No. 2 Eftimie Murgu Square, 300041 Timisoara, Romania; maria.iordache@umft.ro; 8 Multidisciplinary Research Center for Malignant Hemopathies, “Victor Babes” University of Medicine and Pharmacy Timisoara, No. 2 Eftimie Murgu Square, 300041 Timisoara, Romania

**Keywords:** cardio-oncology, cardiotoxicity, machine learning, artificial intelligence, imaging, stereoscopic video capture, SGLT2 inhibitors

## Abstract

**Background/Objectives**: Anthracycline-induced cardiotoxicity remains a major challenge in cancer treatment, and researchers are showing interest in artificial intelligence (AI) to improve the prediction and detection of cancer therapy-related cardiac dysfunction (CTRCD). Current surveillance strategies rely mainly on left ventricular ejection fraction and, more recently, global longitudinal strain. **Methods**: The present study was designed to evaluate cardiac performance in a rat model of doxorubicin-induced cardiotoxicity and empagliflozin-mediated cardioprotection using a machine learning-based analytical framework. Eighteen adult male Sprague–Dawley rats were assigned to five experimental groups. We aimed to quantify ventricular wall dynamics and contractility using an advanced image-processing and object-detection model that has not been previously used to distinguish normal from impaired cardiac kinetics. During real-time recording, simultaneous electrocardiogram monitoring was performed, enabling direct correlation between deep learning-based ventricular wall motion metrics and cardiac electrical activity. The cardioprotective effects of empagliflozin were further validated by immunofluorescence staining (cTnI, vimentin, α-SMA, and Cx43) of rat cardiomyocytes and paraffin-embedded cardiac tissue, demonstrating attenuation of cellular injury and structural remodeling. **Results**: The integrated analysis of cardiac kinetic patterns derived via machine learning distinguishes not only extreme cardiotoxicity, but also tracks a graded pattern consistent with ECG-derived severity and treatment-related functional preservation. These findings indicate that the algorithm captures the gradient of empagliflozin’s cardioprotective effect within this internally validated preclinical setting. Additionally, immunofluorescence results validated the benefits of SGLT2 inhibition on myocardial integrity. **Conclusions**: The novelty of the present work lies at the intersection of advanced cardiac kinetic analysis using AI, preclinical modeling, and SGLT2-mediated cardioprotection in cardio-oncology.

## 1. Introduction

Cancer is one of the global public health challenges. Anthracyclines, such as doxorubicin (DOX), remain a key component of chemotherapy due to their antineoplastic efficacy and ability to reduce tumor progression [1,2]. However, the clinical success of DOX is often limited by its dose-dependent cardiotoxicity, which was initially described in clinical practice as presenting in three distinct patterns. The acute form appears soon after exposure, sometimes even after a single dose, with symptoms within 14 days post-treatment. Early chronic toxicity, which is most common, develops within the first year after treatment and often shows as dilated cardiomyopathy. On the other hand, late chronic toxicity can appear many years or even decades after therapy ends [3]. Recent studies question this timeline, suggesting that anthracycline cardiotoxicity is a continuous pathological process initiated at the level of myocardial cells, progressively impairing contractile function, and eventually leading to heart failure (HF) [4,5].

Anthracycline-induced cardiotoxicity (AIC) has prompted the development of cardio-oncology, a multidisciplinary field focused on optimizing cardiovascular health in patients with cancer [6]. Cardio-oncology combines expertise from cardiology, oncology, hematology, and advanced practice providers to prevent, monitor, and manage cancer therapy–related cardiac dysfunction (CTRCD) [7]. The primary objective is to facilitate the safe administration of oncologic treatments while reducing the risk of both acute and chronic cardiac dysfunction [8,9]. Despite improved cancer treatment protocols, the main cardioprotective drugs for patients receiving anthracyclines: beta-blockers, renin–angiotensin system inhibitors, statins, and dexrazoxane, provide only partial protection and are supported by limited evidence (class IIa recommendation) [8]. This leaves an unmet need for agents that act on the multifactorial mechanisms of injury. AIC is caused by oxidative stress, mitochondrial dysfunction, disrupted calcium handling, topoisomerase II inhibition, endoplasmic reticulum stress, and inflammation [2]. An ideal cardioprotective drug would target several of these pathways simultaneously without compromising the effectiveness of cancer treatment.

Among emerging therapeutic candidates, sodium–glucose cotransporter 2 (SGLT2) inhibitors have drawn increasing attention. Recent evidence suggests that SGLT2 inhibitors have direct cardioprotective effects, reducing myocardial oxidative stress and inflammation, improving mitochondrial energetics, and attenuating fibrosis—mechanisms that directly counter the principal drivers of AIC [10]. Although primarily developed for glycemic control in diabetes, SGLT2 inhibitors are now recommended as a class I treatment for HF, regardless of ejection fraction (EF) [11]. Key clinical trials, such as EMPEROR-Reduced and DAPA-HF, have shown that these drugs reduce HF hospitalization and improve survival when used in combination with guideline-directed medical therapy (GDMT) [12,13]. In these pivotal trials, SGLT2 inhibitors were added on top of GDMT, and their benefit should thus be interpreted as an effect additive to, rather than independent of, optimal GDMT, although the glycemia-independent benefit observed in EMPA-REG OUTCOME [14], a type 2 diabetes population, supported the hypothesis that SGLT2 inhibitors may exert direct cardiac and vascular effects beyond glucose lowering. The cardioprotective effects shown in these trials have led to growing interest in the use of SGLT2 inhibitors for cancer patients [15]. Their pleiotropic actions are especially relevant for patients receiving cardiotoxic cancer therapies, where myocardial injury and functional decline are common features of CTRCD. This statement places SGLT2 inhibitors as promising agents for preventing or attenuating CTRCD [16,17,18].

Preclinical studies have demonstrated that SGLT2 inhibitors attenuate anthracycline-induced cardiac dysfunction by reducing DOX-related increases in circulating injury markers (creatine kinase-MB, cardiac troponin-T) and N-terminal pro-B-type natriuretic peptide (NT-proBNP), consistent with preserved cardiomyocyte integrity and ventricular function [19,20].

At a mechanistic level, the cardioprotective actions of SGLT2 inhibitors in AIC have been linked to modulation of oxidative stress, inflammation, cell death, and metabolic remodeling pathways [21]. Preclinical models have demonstrated that SGLT2 inhibitors reduce myocardial reactive oxygen species (ROS) generation and restore antioxidant defenses (such as superoxide dismutase, catalase, and malondialdehyde acid), thereby neutralizing one of the central mechanisms of DOX-induced cardiac damage [19,22]. Additional studies have implicated pathways such as JNK/Nrf2, AMPK–mTOR, and nucleotide-binding domain-like receptor protein 3 (NLRP3) inflammasome signaling, suggesting that SGLT2 inhibitors exert a broad, pleiotropic influence on the molecular network involved in anthracycline-induced myocardial injury [23,24,25].

Histologically, the cardiotoxic effects of DOX are characterized by vacuolization and loss of striations in cardiomyocytes, inflammation, fibrosis, and cardiomyocyte atrophy. Treatment with SGLT2 inhibitors has been associated with significant attenuation of these microscopic changes, resulting in better preservation of myofibrillar architecture, fewer degenerative changes, and lower semi-quantitative fibrosis scores compared with DOX monotherapy [26,27]. Experimental DOX exposure typically prolongs the QT and QTc intervals, widens the QRS complexes, and produces nonspecific ST-segment alterations, increasing arrhythmia susceptibility [27]. Administration of SGLT2 inhibitors has been reported to attenuate these electrocardiographic abnormalities, suggesting stabilization of ventricular repolarization and conduction [26,27,28].

Other studies have evaluated SGLT2 inhibitor–mediated cardioprotection primarily using echocardiography, demonstrating improvements in left ventricular (LV) function, cardiac strain, and diastolic LV function (lower E/E′) [29,30]. Similar findings have been reported with dapagliflozin (DAPA) in DOX-treated rats, where attenuation of LVEF decline and reduction in cardiac hypertrophy further supported a class effect of SGLT2 inhibitors on global systolic performance [31,32]. In addition to conventional echocardiography, Chang et al. have integrated invasive pressure–volume loop analysis, showing that DAPA pretreatment preserved ventricular volumes and key systolic/diastolic indices in streptozotocin (STZ)–DOX rats, thereby markedly attenuating DOX-induced hemodynamic impairment [33]. Advanced imaging has also been used to complement echocardiography, with cardiac MRI studies showing that SGLT2 inhibitors attenuate DOX-induced LV remodeling, myocardial hypertrophy, and systolic dysfunction in preclinical models [22,34,35].

The present study was designed to evaluate cardiac performance in a rat model of DOX-induced cardiotoxicity and EMPA-mediated cardioprotection using a machine-learning (ML)-based analytical framework. We aimed to quantify ventricular wall dynamics and contractility using an advanced image-processing and object-detection model, developed and validated by our research group, which has not been previously used to distinguish normal from impaired cardiac kinetics. In contrast to prior preclinical work that relied predominantly on non-invasive imaging modalities (e.g., transthoracic echocardiography, GLS, MRI) to assess LV function, our approach is based on real-time visualization of the heart during open-chest surgery under endotracheal intubation and mechanical ventilation. High-resolution three-dimensional (3D) video recordings of the beating heart were acquired to generate a detailed imaging dataset that served as the foundation for training and testing deep learning algorithms for motion analysis and contractility assessment. Through this strategy, our study characterizes the functional impact of DOX and EMPA on cardiac performance and establishes a novel methodological platform for objective, high-fidelity evaluation of cardiac kinetics in experimental cardio-oncology. During real-time recording, simultaneous electrocardiogram (ECG) monitoring was conducted in all rats, allowing direct correlation between deep learning-based ventricular wall motion metrics and cardiac electrical activity.

The alterations in cardiac kinetic performance identified by the machine learning-based analysis were further corroborated with immunofluorescent (IF) staining of cardiomyocyte cultures and myocardial tissue from the same experimental groups, which delineated group-specific patterns of cellular injury and remodeling.

This study introduces a machine learning–driven framework for analyzing cardiac kinetics in a preclinical rat model of DOX-induced cardiotoxicity, thereby extending the role of AI in cardio-oncology beyond conventional imaging and ECG applications.

## 2. Materials and Methods

### 2.1. Animals

The study was conducted on 18 adult male Sprague–Dawley rats (400–450 g) sourced from the Cantacuzino National Research Institute (Bucharest, Romania). Animals were kept under standardized conditions, with a 12 h light/12 h dark cycle, controlled temperature (22 ± 1 °C), and ad libitum access to water and standard laboratory food. All procedures complied with existing ethical regulations and were approved by the Institutional Ethics Committee of “Victor Babeș” University of Medicine and Pharmacy, Timisoara, Romania (approval No. 40; date of approval: 23 July 2025). The study did not involve any human participants or human-derived biological material.

### 2.2. Animal Model of Doxorubicin-Induced Cardiotoxicity

The Sprague–Dawley rats were distributed into five experimental groups following the same cardiotoxicity and treatment protocol previously established and validated by our research group [27]. Briefly, the control group (group I; *n* = 4) received water daily by oral gavage and 0.9% saline injections via intraperitoneal (i.p.) injection at 48 h intervals. The EMPA group (group II; *n* = 3) received EMPA (10 mg/kg/day) daily by oral gavage plus i.p. saline. The EMPA + DOX group (group III; *n* = 3) received EMPA daily, together with DOX (2.5 mg/kg i.p. at 48 h intervals; cumulative dose 15 mg/kg). The DOX group (group IV; *n* = 5) received water daily and the same DOX regimen. For groups I-IV, experiments concluded on day 14. The EMPA preconditioning + DOX group (group V; *n* = 3) underwent 14 days of daily EMPA pre-treatment, followed by a combined EMPA (10 mg/kg/day) and DOX (2.5 mg/kg i.p. at 48 h intervals, cumulative dose 15 mg/kg) protocol. This group allowed assessment of cardioprotective effects of prior EMPA exposure. Total experimental duration for group V: 28 days.

### 2.3. Sample Acquisition

**Figure 1:**
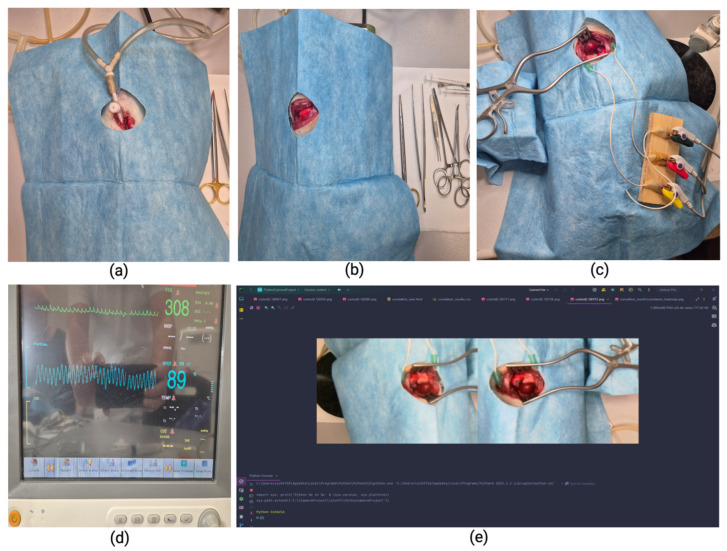
System set-up for the anesthetic-surgical protocol and video recording of the rat hearts. (**a**) The procedure begins with endotracheal intubation of the rat through a tracheostomy. (**b**) A left lateral thoracotomy is performed to expose the heart. (**c**) A small thoracic retractor is used to improve visualization of the heart. The heart is carefully separated from the lung tissue to minimize movement during video recording. Subcutaneous electrodes are attached to monitor the ECG throughout the procedure. (**d**) A screenshot of the monitor displaying continuous ECG and oxygen saturation readings during a session. (**e**) Frame split view of real-time heart recording using OpenCV(v4.12.0) in Python (v3.12.0).

The Sprague–Dawley rats were placed on a heated operating table set to 36 °C to prevent hypothermia and preserve stable physiological conditions. General anesthesia was induced with isoflurane (5%) and maintained at 2–2.5% in oxygen. After induction of anesthesia, tracheostomy was performed, and a 14-G peripheral venous catheter was inserted into the trachea for controlled mechanical ventilation. A left lateral thoracotomy was performed, carefully opening the chest. The pericardium was located, incised, and retracted to expose the heart, especially the left ventricle (LV). To monitor heart electrical activity, electrodes were attached, and the electrocardiogram (ECG) was recorded throughout the procedure using a CONTEC CMS6000 monitor (Contec Medical Systems Co., Ltd., Qinhuangdao, Hebei, China). Real-time quantification of ventricular wall dynamics and contractility in the open-thorax model was provided by developing a non-contact optical acquisition system coupled with a computer vision pipeline. High-fidelity video data of the beating heart was acquired using a synchronized dual-lens stereoscopic camera system (ELP Dual Sync USB, ELP, Shenzhen, China) (Figure 1).

**Figure 2:**
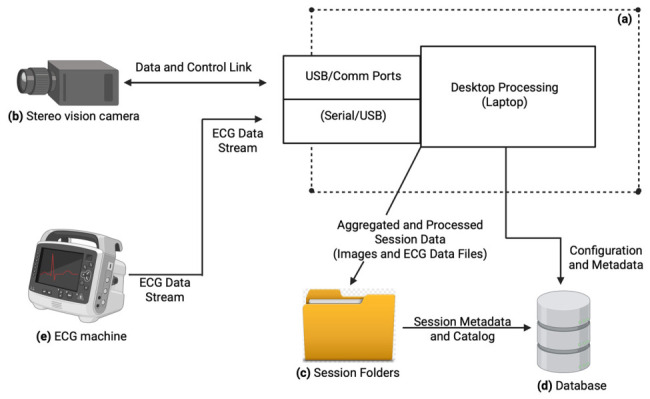
The monitoring system is composed of the following elements: (**a**) desktop module (laptop); (**b**) stereo vision camera; (**c**) session folders; (**d**) database; (**e**) ECG machine (CONTEC monitor).

After thoracotomy, hemodynamic stabilization was achieved, during which the stereoscopic video camera position was adjusted to obtain high-quality images of the beating heart in situ. Subsequently, the recording phase was initiated, with continuous image capture and electrocardiographic signal acquisition for a standardized 5 min period per animal. The configuration of the monitoring system is illustrated in Figure 2.

### 2.4. Electrocardiographic Data

ECG data were captured directly from the monitor’s Ethernet output to maximize signal quality, thereby avoiding data loss associated with analog–to–digital conversion. Raw network traffic was recorded with Wireshark (v4.6.3) and saved as packet capture (.pcap) files, from which the binary ECG stream was extracted and converted into an ASCII coordinate format (.asc) representing voltage over time. The resulting signals were processed using the open-source ecg_processing framework (WFDB Python package, version 4.3.0, GitHub) to filter out baseline noise, identify R peaks, and isolate individual P–QRS–T complexes for further analysis, following the same acquisition and processing pipeline as in our previous study [27]. All ECGs were reviewed by cardiologists from our research team with experience in chemotherapy-related cardiotoxicity. The ECG assessment included evaluation of heart rate (HR), PR and QT intervals, and QRS duration. The corrected QT interval (QTc) was derived using the Bazett formula. In addition, ST-segment and T-wave morphology indicative of DOX-related myocardial injury were systematically evaluated.

### 2.5. Cardiac Performance Assessment Using Machine Learning Techniques

The analysis pipeline consists of four sequential stages applied uniformly to all rat hearts across the five treatment groups. Each stage is described below:

#### 2.5.1. Video Acquisition—Proprietary Stereo Capture Software

The rat hearts were imaged using a custom dual-camera stereoscopic system, producing a synchronized 1280 × 480 px side-by-side stream at 60 frames per second (fps), delivering sufficient sampling resolution to capture the fast cardiac cycle of the murine model. Stereo geometry was established via a 9 × 6 checkerboard calibration (OpenCV stereoCalibrate). Intrinsic and extrinsic parameters were stored as NumPy.npz archives and applied to every recording session. Each 50 s acquisition yielded 3000 depth frames at a resolution of 384 × 384 pixels (px) per heart. Proprietary capture software handled camera synchronization, rectification-map application, and real-time preview. All raw frames were written to disk for offline processing.

#### 2.5.2. Heart Segmentation—SAM2 (Segment Anything Model 2)

The heart region was automatically detected and delineated in each RGB frame using SAM2 (Meta AI). A single-point prompt at the frame center initialized the segmentation mask, which SAM2 then propagated across all 3000 frames, robustly tracking the heart boundary through contractile motion and slow positional drift due to perfusion-pressure oscillations. The propagated masks served two purposes: (i) they constrained all downstream depth analysis exclusively to heart tissue, excluding the surrounding connective tissue; and (ii) a time-averaged binary mask—thresholded at the 10th percentile of mean-frame depth values, then expanded by a 3-pixel morphological dilation—was applied uniformly to all frames, with non-heart pixels set to NaN. The resulting isolated depth time series were saved as NumPy float32 arrays.

#### 2.5.3. Depth Estimation and Point Cloud Construction Using MiDaS

**Figure 3:**
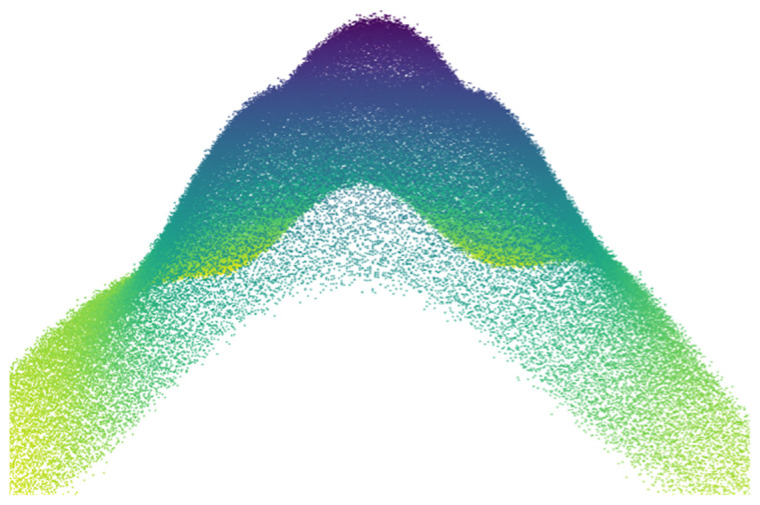
Depth visualization. For visualization, the depth axis was inverted, causing the heart to be displayed as a smooth dome emerging from a flat plane. Lighter green tones indicate structures located farther from the camera, whereas darker blue tones indicate structures closer to the camera.

**Figure 4:**
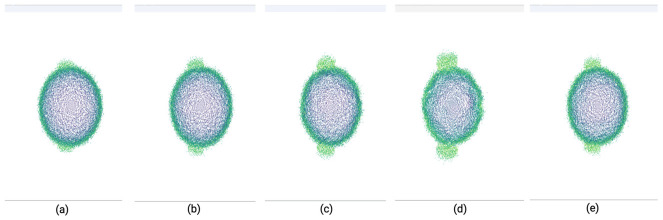
Isolated heart point cloud top-down view. (**a**) Control (group I); (**b**) EMPA (group II); (**c**) EMPA + DOX (group III); (**d**) DOX (group IV); (**e**) EMPA-pre + DOX (group V). Lighter green tones indicate structures located farther from the camera, whereas blue tones indicate structures closer to the camera.

Monocular depth maps for each frame were estimated from the left-camera RGB images using MiDaS v2.1 Small (midas_v21_small_256.onnx, ONNX Runtime with CUDA). MiDaS provides inverse relative depth, so higher values indicate regions closer to the lens. The heart, which is the closest structure, consistently has the lowest depth values in each frame. Each frame was converted to a 3-D point cloud by assigning pixel (x, y) coordinates to the corresponding depth value z, scaled by 200 for spatial proportionality. For visualization, the depth axis was inverted so that the heart appears as a smooth dome rising above a flat plain (Table 1; Figure 3). The apex of the dome oscillates in height at the cardiac frequency of ~5 Hz (approximately 300 beats per minute in control hearts). A Fourier-domain bandpass filter was used to remove slow perfusion-pressure drift (0.1 to 4.0 Hz per pixel) while keeping the full cardiac frequency band above 4 Hz (Figure 4).

#### 2.5.4. Health Scoring and ECG Correlation

The cardiotoxicity score was derived in a fully unsupervised, data-driven manner, using the depth-camera recordings of the beating heart exclusively. No labeled training data, no ECG information, and no group assignment were used at any stage of its construction. For each subject (up to 120 frames), valid pixels were projected into a 3-D space (x and y from the pixel indices; z from the depth value multiplied by 200) and clustered per frame with DBSCAN (ε = 8.0, min_samples = 15). Five feature classes were extracted per frame: (i) noise ratio (fraction of DBSCAN-noise points), (ii) cluster count, (iii) mean cluster size, (iv) intra-cluster compactness (mean pairwise Euclidean distance within each cluster), and (v) fragmentation index (cluster count/total point count). These were aggregated over time by their temporal mean and standard deviation, yielding nine subject-level features (a mean and a standard deviation for the first four descriptors, plus the mean fragmentation index). Conceptually, the mean features quantify the average degree of spatial organization of the contractile motion, whereas the standard-deviation features quantify its beat-to-beat temporal stability; both are expected to deteriorate under cardiotoxic injury. The resulting matrix (18 subjects × 9 features) was standardized column-wise via a Z-score transformation, placing all features on a common, dimensionless scale so that each contributes equally to the composite score, irrespective of its original units or magnitude. The features were then sign-harmonized so that a positive Z-score uniformly denotes a sicker state—important cardiotoxicity (only the mean cluster size, which is higher in healthier hearts, was inverted). For each subject, the sign-harmonized Z-scores were averaged across the nine features to obtain a single composite index (C_i_), where a higher value indicates a sicker animal (i.e., greater cardiotoxicity) across all feature dimensions simultaneously. Finally, the composite indices were linearly rescaled onto an interpretable 1.0–10.0 interval by min–max normalization, score_i_ = 1 + 9 × (C_i_ − C_min_)/(C_max_ − C_min_), mapping the least affected animal of the cohort to 1.0 and the most affected to 10.0, with the remaining animals placed proportionally in between. The resulting scale is cohort-relative, providing an ordinal ranking of severity.

To ensure methodological symmetry, the composite ECG severity index used for cross-validation was constructed using an independent, parallel pipeline that applied the same Z-score normalization, sign harmonization, and min–max rescaling to the six ECG metrics (QRS, PR, and QT intervals; R-wave, T-wave, and HR values). The two scoring systems were therefore built by the same procedure but from entirely independent data streams—optical point clouds on the one hand and ECG waveforms on the other.

Because the murine cardiac cycle (~5 Hz, ~300 bpm) is considerably faster than the human one, several steps were implemented to suppress baseline drift and motion artifacts: (i) acquisition at 60 fps satisfied the Nyquist criterion for the cardiac frequency band; (ii) SAM2-propagated masks restricted the analysis to the myocardial surface, excluding the motion of surrounding tissue; (iii) per-pixel Fourier-domain band-pass filtering removed the slow perfusion-pressure and residual respiratory drift while retaining the full cardiac band above 4 Hz; and (iv) the DBSCAN noise-ratio feature explicitly quantified residual non-clustered (artefactual) points, so that such points were incorporated into the score rather than confounding it.

#### 2.5.5. Statistical Analysis of Point Cloud Cardiotoxicity Score and ECG Metrics

Continuous variables were presented as mean ± standard deviation. The relationship between the point cloud cardiotoxicity score and ECG parameters was evaluated across the full cohort of 18 animals, with a single point cloud value and a single ECG value per animal. Seven correlations were tested: the point cloud score versus each of the six individual ECG metrics (QRS duration, PR interval, QT interval, R-wave amplitude, T-wave amplitude, and HR) and versus the composite ECG severity index. For every pair, both the Pearson coefficient (r), assessing the linear association, and the Spearman rank coefficient (ρ), a rank-based and distribution-free measure, were computed. The normality of each variable was assessed with the two-sided Shapiro–Wilk test (α = 0.05); when a variable in a pair deviated from normality (Shapiro–Wilk *p* < 0.05), the Spearman coefficient was taken as the primary statistic, whereas Pearson’s r was used as the primary statistic when both variables were normally distributed. 95% confidence intervals for all coefficients were obtained using Fisher’s z-transformation. To control the false-discovery rate across the seven comparisons, the Benjamini–Hochberg procedure was applied separately to the Pearson and the Spearman *p*-values, with a corrected significance threshold of q < 0.05. Statistical significance was set at *p* < 0.05 (two-sided). Analyses were performed using Python (v3.12.0).

### 2.6. Isolation and Culture of Rat Cardiomyocytes

**Figure 5:**
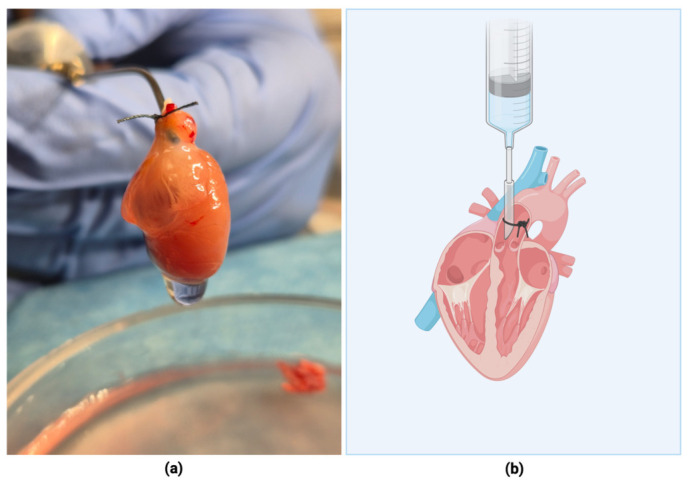
Cannulation of the adult rat heart. (**a**) The rat heart was cannulated via the aorta and connected to a syringe attached to the cannula. (**b**) Schematic illustration depicting the cannula’s position within the aorta in relation to the aortic valve and the suture tie. The cannula should be inserted into the ascending aorta, taking care to avoid crossing the aortic valve. Created in BioRender (https://BioRender.com/ml08lou (accessed on 18 June 2026)).

Prior to heart harvesting, the inferior vena cava was exposed, and systemic heparinization was achieved with unfractionated heparin (Pan Pharma GmbH, La Selle-en-Luitré, France) at a dose of 3.0 IU/g. This step was performed to prevent blood coagulation and embolism within coronary circulation. Hearts were rapidly excised at the end of video recording and cannulated at the level of the ascending aorta. After cannulation, the hearts were washed with collagenase solution (10 mg/mL in PBS; Collagenase from Clostridium histolyticum, type I A, Sigma-Aldrich, St. Louis, MO, USA, C9891-100 mg) supplemented with 36 mL of Calcium chloride (CaCl_2_; 100 mM; Sigma-Aldrich) for 20–30 min (Figure 5). The hearts were then transferred to 10 cm Petri dishes containing Dulbecco’s phosphate-buffered saline (DPBS; Gibco, ThermoFisher Scientific, Waltham, MA, USA) supplemented with 1% Penicillin–Streptomycin (Pen Strep; Gibco).

Emergent blood vessels and residual connective tissue were carefully removed using a scalpel and forceps. The ventricular tissue was transferred to clean 10 cm Petri dishes. The tissue was cut into small fragments and incubated with 15 mL 0.05% Trypsin-EDTA (0.05% Trypsin-EDTA (1×); Gibco) in a humidified cell culture incubator (37 °C, 5% CO_2_) for approximately 30 min. Enzymatic digestion was stopped by adding 15 mL of Trypsin stop solution consisting of RPMI 1640 medium (1×; Gibco) supplemented with 10% FBS Qualified (Fetal Bovine Serum; Gibco). The resulting cell suspension (approximately 30 mL) was distributed into three 15 mL conical tubes (Falcon 15 mL High Clarity PP Centrifuge Tube; Corning)—10 mL/each tube.

25 µL CaCl_2_ (CaCl_2_ solution, concentration of 100 mM) was added to each tube three times at 5 min intervals, followed by a final addition of 50 µL CaCl_2_ before centrifugation at 100× *g* for 1 min. After centrifugation, the supernatant was removed, and the sediment was resuspended in 2 mL culture medium composed of DMEM/F-12 (Gibco) supplemented with 10% FBS, 1% Pen Strep, and 200 µL ITS (ITS Liquid Media Supplement 100×; Sigma) and placed in the cell incubator for 24 h. The medium was then replaced with serum-free maintenance medium.

Cells were seeded in Poly-L-lysine–coated 24-well plates (CELLCOAT Poly-L-Lysine, 24-well, PS, clear, lid with condensation rings) and cultured for an additional 24 h in a humidified incubator (37 °C, 5% CO_2_).

### 2.7. Immunofluorescence Staining

#### 2.7.1. Rat Cardiomyocytes

A subset of rat heart-isolated cells was removed from the culture plate 10 days after isolation and was submitted to immunofluorescence staining. 250 µL of cellular suspension was cytocentrifuged using Cytofunnel-Cytoslides ensemble (EZ Single Cytofunnel, white—Microscope Slides for Cytospin, coated; Epredia, Runcorn, UK) and Shandon Cytospin 4 (Thermo Scientific, Runcorn, UK) for 6 min at 600 rpm. The cells were then fixed in 4% paraformaldehyde for 10 min at room temperature, washed, and stained overnight at 4 °C with the following primary antibodies: anti-α-smooth muscle actin (rabbit anti-α-SMA; ab32575, Abcam, Cambridge, UK), anti-cardiac troponin I (rabbit anti-cardiac troponin I; ab47003, Abcam), and anti-vimentin (goat anti-vimentin, R&D Systems, Minneapolis, MN, USA). The anti-connexin 43 monoclonal antibody (mouse anti-Cx43, clone CX-1B1, Invitrogen, Carlsbad, CA, USA) was already conjugated to a fluorochrome (Alexa Fluor 488). The immunofluorescence staining continued with the secondary antibody, goat anti-rabbit AlexaFluor 594 (Invitrogen) for α-SMA and Troponin I, while the anti-vimentin antibody was conjugated with a donkey anti-goat AlexaFluor 546 (Invitrogen). After 30 min in the dark, at room temperature, the cytoslides were washed twice with DPBS (Gibco) and counterstained for the nuclei using DAPI (4′,6-diamidino-2-phenylindole; ThermoFisher Scientific, Waltham, MA, USA) solution (10 mg/mL) for 5 min. The slides were visualized, and images were acquired using an EVOS FL Auto 2 fluorescence microscope (ThermoFisher Scientific) under identical exposure settings across experimental groups.

#### 2.7.2. Paraffin-Embedded Cardiac Tissues

Immunofluorescence (IF) staining was performed on formalin-fixed, paraffin-embedded ventricular tissue sections to further characterize structural and phenotypic alterations associated with anthracycline-induced myocardial injury. The expression and distribution of α-SMA, cTnI, vimentin, and Cx43 were evaluated using specific primary antibodies and fluorescent-labeled secondary antibodies (as described in the previous section) according to standard immunofluorescence protocols. A 10 mg/mL DAPI solution was used for nuclear staining. Sections were examined by fluorescence microscopy (Evos FL Auto 2, Invitrogen, ThermoFisher Scientific, Carlsbad, CA, USA).

#### 2.7.3. Statistical Analysis of Immunofluorescence Data

Immunofluorescence images were quantitatively analyzed using ImageJ (version 1.53t) by an experienced investigator blinded to the experimental condition, under identical acquisition settings. Individual fluorescence channels were separated prior to analysis, and background fluorescence was determined by measuring the mean fluorescence intensity in cell-free or tissue-free regions of each image, then subtracting it from the corresponding fluorescence signal. For each experimental condition, eight representative microscopic fields were analyzed under identical image acquisition and processing settings. Regions of interest (ROIs) were manually defined, and fluorescence measurements were obtained as integrated density and area. The mean fluorescence intensity (MFI) was calculated as the ratio of integrated density to the corresponding area and expressed in arbitrary units (A.U.). Quantitative data are presented as mean ± standard deviation (SD). Statistical analyses were performed using GraphPad Prism (version 7.0). Each marker was analyzed independently by one-way ANOVA. Comparisons between treatment groups and the corresponding control group were performed using Dunnett’s multiple comparisons test, and differences were considered statistically significant at *p* < 0.05.

## 3. Results

### 3.1. ECG Findings

In our previous model of anthracycline cardiotoxicity [27], DOX administration led to clear ECG changes, including slowed conduction and abnormal repolarization. Compared to control rats, those in the DOX group (group IV) had a much lower heart rate and longer QRS, PR, QT, and QTc intervals. These changes suggest problems with atrioventricular conduction, slower ventricular depolarization, and prolonged overall ventricular repolarization. The R-wave amplitude also decreased significantly, and the T-wave became negative. These outcomes are typical of heart muscle injury and repolarization abnormalities seen in DOX cardiotoxicity models.

**Figure 6:**
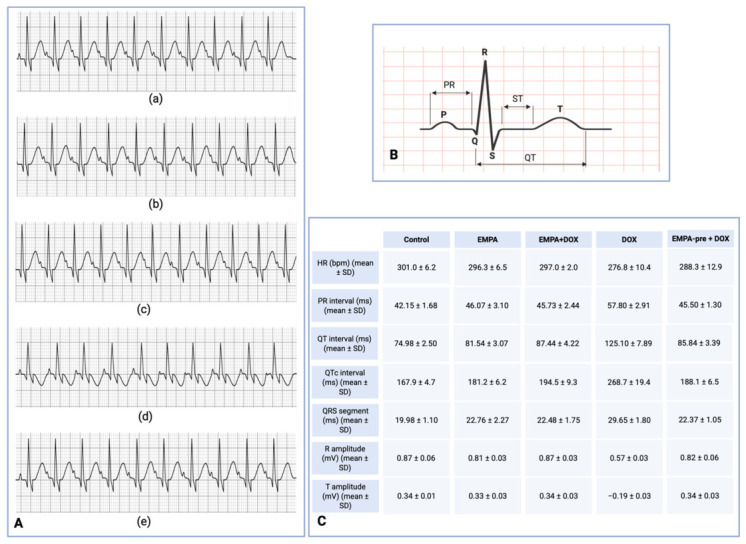
Electrocardiographic comparisons across the experimental groups. (**A**) (**a**) Control group, with QRS, PR, QT, and QTc intervals and R- and T-wave amplitudes within normal limits. (**b**) The EMPA group showed an ECG pattern comparable to that of the control group. (**c**) EMPA + DOX group, in which EMPA attenuated DOX-induced prolongation of QRS, PR, and QTc intervals and largely preserved R- and T-wave amplitudes. (**d**) DOX group, displaying reduced HR, widening of QRS, PR, QT, and QTc intervals, and characteristic changes in R- and T-wave amplitudes typical of DOX-induced cardiotoxicity. (**e**) EMPA-pre + DOX group in which EMPA pretreatment similarly limited QRS, PR, QT, and QTc prolongation and preserved R- and T-wave amplitudes compared with the DOX group. Paper speed: 60 mm/s; amplitude calibration: 10 mm/mV. (**B**) Schematic representation of ECG waves, intervals, and segments. (**C**) ECG measurements for all groups.

Empagliflozin treatment significantly reduced this electrophysiological deterioration. In both EMPA-treated groups (III and V), QRS, PR, QT, and QTc intervals were significantly shorter than in DOX monotherapy (group IV) and approached the values observed in the control (group I). Preservation of R-wave amplitude and a positive T wave indicates that EMPA ameliorated DOX-induced impairment of ventricular depolarization and repolarization, restricting the development of a pro-arrhythmic substrate. ECG responses were similar between concomitant and pretreatment empagliflozin regimens (groups III and V), suggesting comparable cardioprotective effects in both approaches, without additional benefit from preconditioning as reflected in ECG parameters. These electrocardiographic changes and the between-group comparisons are more clearly visualized in Figure 6.

Overall, these results support the use of rat ECG as a sensitive measure of anthracycline-induced cardiotoxicity (AIC) and demonstrate that EMPA provides significant cardio-protection.

### 3.2. Machine Learning Signal Classification for Enhanced Detection of Doxorubicin-Induced Cardiotoxicity in Rats

**Figure 7:**
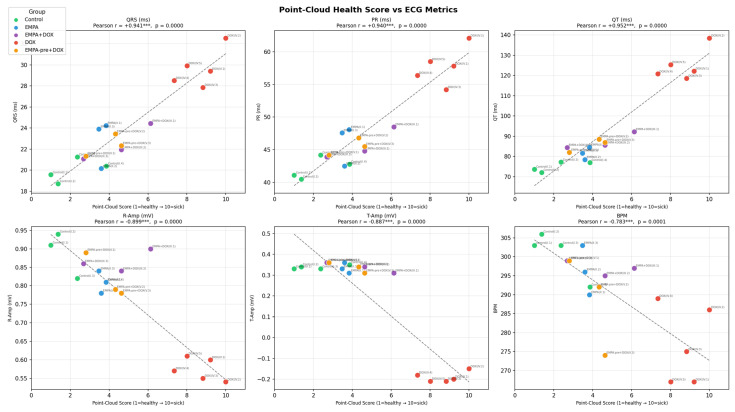
Multi-parametric internal correlation between ECG indices and the point cloud cardiotoxicity score within the same experimental cohort. Each panel depicts the linear regression (Pearson’s r) between the computer-vision-derived cardiotoxicity score (*X*-axis, scale 1.0–10.0) and specific electrophysiological markers (*Y*-axis). Panel 1 displays QRS duration (ms), Panel 2 shows PR interval (ms), Panel 3 presents QT interval (ms), Panel 4 illustrates R-wave amplitude (R-Amp, mV), Panel 5 shows T-wave amplitude (T-Amp, mV), and Panel 6 displays Heart Rate (BPM). Each data point represents an individual subject across the five experimental groups. Statistical significance is indicated as *p* < 0.001 for all correlations (*** = *p* < 0.001).

To evaluate the performance of the unsupervised point cloud cardiotoxicity score in this preclinical setting, we compared it with standard ECG markers of AIC. We found that the contractile motion of the epicardial surface closely matches the heart’s electrical activity. The toxicity score was strongly associated with longer QRS, PR, and QT intervals, which are markers of conduction delays and repolarization abnormalities in AIC. Notably, the QT interval, a primary marker of cardiotoxicity, showed the strongest alignment with point cloud metrics, confirming that spatial fragmentation and noise in the point cloud are direct indicators of electrical instability. We also found strong negative correlations between the toxicity score and R-wave and T-wave amplitudes (R-Amp and T-Amp), as well as heart rate (BPM). Lower voltage amplitudes suggest a loss of healthy heart muscle and weaker electrical signals, which the algorithm detected as fewer compact clusters and a higher fragmentation index. These findings highlight the potential of 3D point cloud analysis as a promising experimental marker for cardiac monitoring, effectively mapping the transition from healthy electromechanical coupling to the fragmented, bradycardic phenotype characteristic of severe DOX-induced HF (Figure 7).

**Figure 8:**
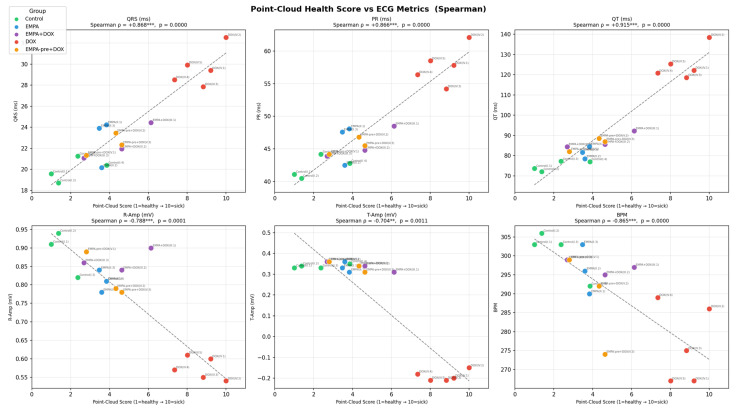
Internal non-parametric Spearman rank-order correlations between the cohort-relative point cloud cardiotoxicity score and ECG parameters in the same experimental cohort. The multi-panel scatter plots show a monotonic relationship between the unsupervised optical severity score on the *X*-axis (graded 1.0–10.0) and electrophysiological metrics on the *Y*-axis. Panel 1 displays QRS duration (ms), Panel 2 shows PR interval (ms), Panel 3 presents QT interval (ms), Panel 4 illustrates R-wave amplitude (R-Amp, mV), Panel 5 shows T-wave amplitude (T-Amp, mV), and Panel 6 displays Heart Rate (BPM). All variable pairs analyzed are statistically significant at *p* < 0.001 (** = *p* < 0.01, *** = *p* < 0.001).

To further investigate whether the point cloud cardiotoxicity score represents an internally consistent preclinical signal, we employed a non-parametric Spearman rank-order correlation analysis alongside the linear models. While Pearson’s test established the general trajectory of cardiotoxicity, Spearman’s coefficient is crucial from a biostatistical perspective for confirming the monotonic progression of myocardial dysfunction across distinct ordinal stages of severity. As shown in Figure 8, the optical point cloud score demonstrated highly significant, positive monotonic correlations with the QRS, PR, and QT intervals. This finding confirms that as the computer-vision algorithm assigns a higher rank to structural fragmentation and kinematic noise, there is a consistent, stepwise deterioration in atrioventricular conduction and ventricular repolarization. Negative Spearman correlations were observed for R-wave and T-wave amplitudes, as well as HR. The consistency of these non-parametric associations is vital. It demonstrates that the automated spatial-kinematic scoring system not only differentiates extreme toxicity from healthy controls but also tracks a graded pattern consistent with ECG-derived severity and treatment-related functional preservation, confirming that the algorithm may help grade the cardioprotection offered by EMPA. Ultimately, these results validate the 3D point cloud model as a statistically stable, ECG-correlated ordinal index for in vivo staging of AIC.

**Figure 9:**
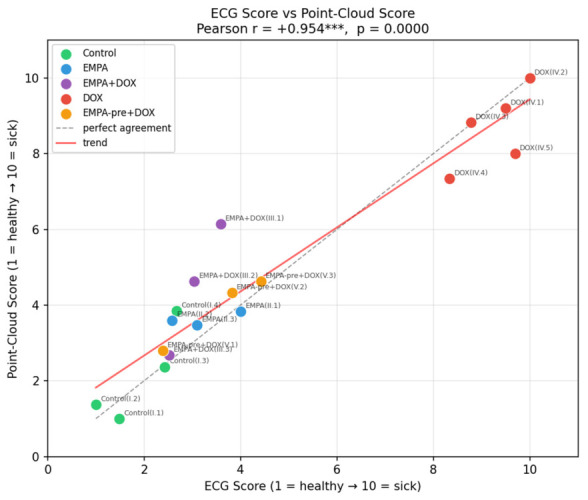
Internal validation of the cohort-relative point cloud cardiotoxicity score using the normalized composite ECG index from the same experimental cohort. To establish a direct 1:1 comparative baseline, specific ECG parameters (QRS, PR, QT, R-Amp, T-Amp, and BPM) were mathematically integrated and normalized into a 1.0-to-10.0 severity scale, matching the optical point cloud scoring system. Linear regression analysis (Pearson’s r = 0.954, *p* < 0.001) demonstrates a highly robust positive relationship between mechanical fragmentation and global electrophysiological decline; (*** = *p* < 0.001).

We created a normalized Composite ECG Index to summarize electrophysiological data and serve as a reference standard for our optical tracking system within this proof-of-concept experimental framework. This index combines conduction and repolarization variables (QRS, PR, QT, R-AMP, T-AMP, and BPM) for each group and scales the results to a 1.0–10.0 scale, making them directly comparable to the unsupervised point cloud cardiotoxicity score. The linear regression model (Pearson r = 0.954, *p* < 0.001) shows a strong, positive relationship: as spatial-kinematic uncoupling increases by 1 unit in the SAM2-driven algorithm, global electrical instability predictably rises (Figure 9).

**Figure 10:**
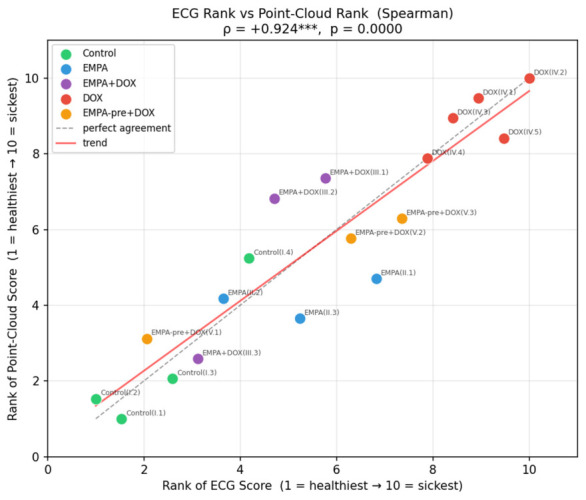
Internal non-parametric validation of the cohort-relative point cloud cardiotoxicity score using the normalized composite ECG index from the same experimental cohort. The scatter plot illustrates the Spearman rank-order correlation (*p* = 0.924, *p* < 0.0001) between the global ECG severity rank (*X*-axis) and the unsupervised spatial-kinematic score (*Y*-axis), both harmonized to a 1.0–10.0 scale. The high monotonic alignment supports the algorithm’s ability to stage the functional ranking across groups: from the healthy baseline (Control/EMPA), through the gradient of therapeutic efficacy (EMPA + DOX and EMPA-pre + DOX), to the severe AIC phenotype (DOX group); (*** = *p* < 0.001).

The Spearman rank-order correlation (*p* = 0.924, *p* < 0.001) also indicates that cardiotoxicity progresses consistently (Figure 10). The algorithm correctly identified the severity order among the experimental subjects, tracking the shift from the healthy control group, through the protected groups (EMPA + DOX and EMPA-pre + DOX), to the severe HF phenotype (DOX group).

**Figure 11:**
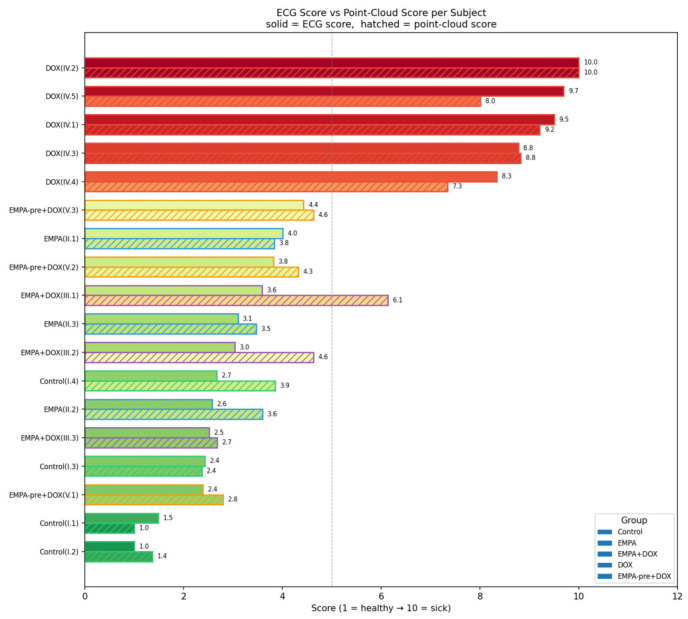
ECG-derived health scores (solid bars) vs. point cloud-derived scores (hatched bars) for all 18 subjects in the same experimental cohort. The unsupervised point cloud scoring assigned scores from 1.0 to 10.0. All four control hearts scored below 4.0. All five doxorubicin hearts (group IV) scored above 7.3. Groups III (EMPA + DOX) and V (EMPA-pre + DOX) occupied intermediate ranges, reflecting partial cardioprotection.

This correlation plot (Figure 11) summarizes the study’s main findings by comparing the Composite point cloud score, based on cluster kinematics and spatial fragmentation, with the Composite ECG Index, which combines QRS, PR, QT intervals, HR, and R/T amplitudes. Both scores are shown on a standard 1.0-to-10.0 severity scale. The strong linear relationship (Pearson’s r = 0.954) and consistent trend (Spearman’s ρ = 0.924) show a direct link between electrical changes and mechanical decline. Treatment with EMPA (groups III and V) keeps the heart near the healthy range and prevents the shift toward the high-toxicity area seen in the DOX group (group IV).

The point cloud cardiotoxicity score was normally distributed (Shapiro–Wilk, *p* = 0.168), whereas five of the six individual ECG metrics and the composite ECG index were not (Shapiro–Wilk, *p* < 0.05). The Spearman coefficient was therefore adopted as the primary statistic for those comparisons. The strongest individual associations involved the conduction and repolarization-related metrics: QT interval (Spearman ρ = +0.915, 95% CI 0.783–0.968), QRS duration (Pearson r = +0.941, 95% CI 0.845–0.978), and PR interval (Spearman ρ = +0.866, 95% CI 0.671–0.949), all *p* < 0.001. Strong negative correlations were observed for R-wave amplitude (ρ = −0.788, 95% CI −0.917 to −0.507), HR (ρ = −0.865, 95% CI −0.949 to −0.668), and T-wave amplitude (ρ = −0.704, 95% CI −0.881 to −0.353), consistent with the loss of viable myocardium, the relative bradycardia, and the repolarization abnormalities that characterize DOX-induced cardiotoxicity. Most importantly, the point-cloud score showed near-complete agreement with the independently derived composite ECG severity index (Spearman ρ = +0.924, 95% CI 0.803–0.972; Pearson r = +0.954, 95% CI 0.879–0.983; both *p* < 0.001). All seven comparisons remained significant after Benjamini–Hochberg correction for multiple testing (q < 0.05).

### 3.3. Rat Cardiomyocytes in Culture

**Figure 12:**
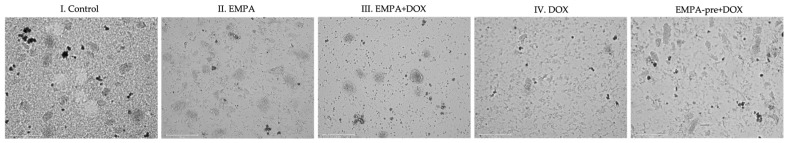
Representative phase-contrast images of rat cardiomyocytes from each experimental group. Cell morphology and overall structural integrity under the different treatment conditions.

Cardiomyocytes were isolated from the left ventricles of rats using a collagenase and 0.05% trypsin dissociation method. There were no statistically significant differences in the general isolation parameters between the experimental groups. Still, the yield of isolated cardiomyocytes was consistently lower in DOX-treated hearts than in untreated controls. This result may indicate fewer viable cardiomyocytes in the myocardium after DOX exposure, but it could also indicate that the cells were more fragile and more easily damaged during enzymatic dissociation. Supporting this idea, many cardiomyocytes from DOX-treated hearts showed signs of deterioration, including membrane instability and loss of intracellular content, which likely led to greater cell disintegration and reduced recovery during isolation (Figure 12).

### 3.4. Immunofluorescence Findings

**Figure 13:**
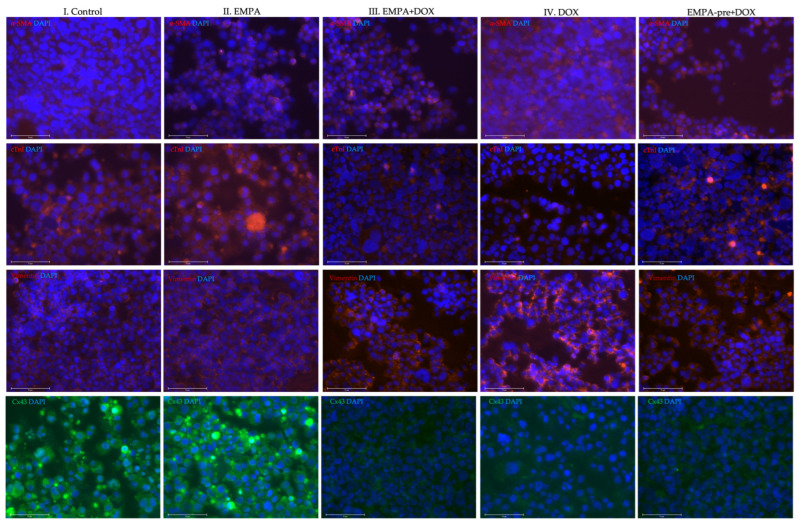
Representative IF images of rat cardiomyocytes isolated from all five experimental groups and stained 10 days after isolation with α-SMA, cTnI, vimentin, and Cx43. DAPI staining was used to label cell nuclei in blue. In the control group (group I), cTnI showed a preserved, homogeneous sarcomeric pattern, Cx43 exhibited a junctional distribution, and α-SMA and vimentin signals were present, but consistent with low fibroblast/myofibroblast activation. The EMPA group (group II) displayed a similar pattern. In group IV (DOX), cTnI staining appeared attenuated and more disorganized, Cx43 signal was reduced or redistributed, and α-SMA and vimentin immunoreactivity were increased, indicating cardiomyocyte injury and stromal activation. Cardiomyocytes from the EMPA + DOX (group III) and EMPA-pre + DOX (group V) showed partial preservation of cTnI and Cx43 patterns and lower α-SMA/vimentin signal compared with group IV. Scale bar: 75 µm.

Immunofluorescence (IF) staining was used to assess the expression and distribution of α-SMA, vimentin, cTnI, and Cx43 in cells from the rat LV. cTnI staining confirmed that the isolated cells were cardiomyocytes, showing positive cytoplasmic expression. In the DOX groups, the cTnI signal was weaker and more disorganized, consistent with AIC features. Groups III (EMPA + DOX) and V (EMPA-pre + DOX) had a more preserved cTnI pattern than group IV, suggesting that SGLT2 inhibition helps protect sarcomeric structure from anthracycline-induced damage. Cx43 was detected predominantly at sites of intercellular contact, consistent with gap junction localization. In DOX-treated groups, Cx43 expression was lower and less evenly distributed compared to the control (group I) and EMPA group (group II). Groups III and V also showed a slightly better Cx43 pattern than group IV (Figure 13).

Vimentin-positive cells, corresponding mainly to fibroblast-like populations, were identified in all groups, while α-SMA expression was observed in a subset of cells suggestive of activated fibroblasts/myofibroblasts or vascular-associated cells. In groups that received DOX, we observed a higher proportion of vimentin-positive cells, which was partially explained by fibroblasts being more resistant than cardiomyocytes in long-term culture but also in the context of anthracycline cardiotoxicity. Also, DOX-associated injury may further favor fibroblast activation and reparative remodeling signals, as evidenced by increased α-SMA-positive staining (Figure 13).

**Figure 14:**
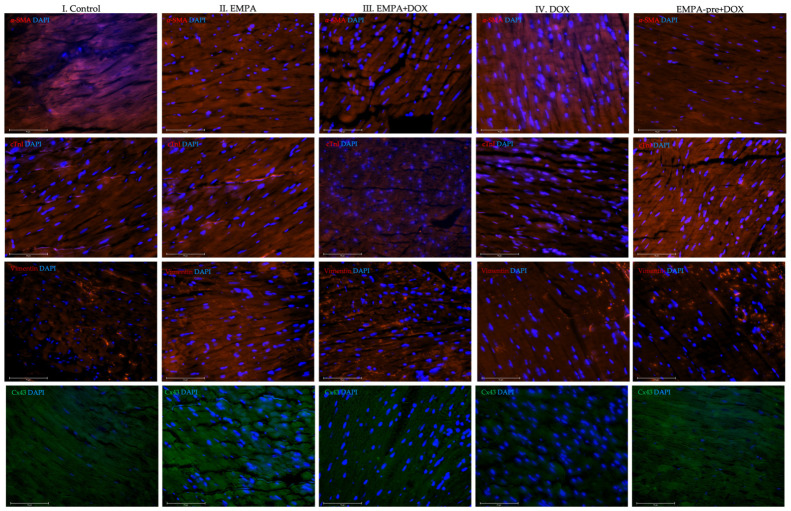
Representative IF images of myocardial tissue from all five experimental groups. Staining of structural cardiomyocyte proteins (cTnI, Cx43) and connective tissue markers (α-SMA, vimentin). DAPI staining was used to label cell nuclei in blue. In group IV (DOX), we observed increased areas of α–SMA–positive myofibroblasts and vimentin-positive interstitial cells, compared with the other groups. Also, in group IV (DOX), loss or patchy reduction in sarcomeric cTnI signal, along with disorganization of Cx43, can be observed. In groups III (EMPA + DOX) and V (EMPA-pre + DOX), these doxorubicin-induced changes were attenuated. Scale bar: 75 µm.

The IF analysis of cardiac tissue revealed variable degrees of myocardial remodeling and cellular injury. In control myocardial tissue (group I), cTnI expression showed homogeneous cytoplasmic staining in cardiomyocytes, while Cx43 demonstrated a regular punctate distribution localized predominantly at intercalated discs, consistent with preserved myocardial electrical coupling. α-SMA immunoreactivity was restricted mainly to vascular smooth muscle cells, with minimal interstitial staining, whereas vimentin expression was limited to resident stromal and endothelial cells (Figure 14).

In DOX-exposed myocardial sections, alterations in marker expression and distribution were observed with variable severity. cTnI staining intensity was reduced and appeared discontinuous in damaged cardiomyocytes, consistent with contractile apparatus disruption and myofibrillar degeneration. Cx43 expression showed partial loss of membrane-associated organization and decreased signal intensity, suggestive of impaired gap junction integrity and electrical remodeling. Increased interstitial α-SMA expression was observed in areas of fibrotic remodeling, indicating the activation of myofibroblast-like cells. Vimentin immunoreactivity was also increased within interstitial regions, reflecting stromal activation and tissue remodeling processes associated with AIC (Figure 14).

**Figure 15:**
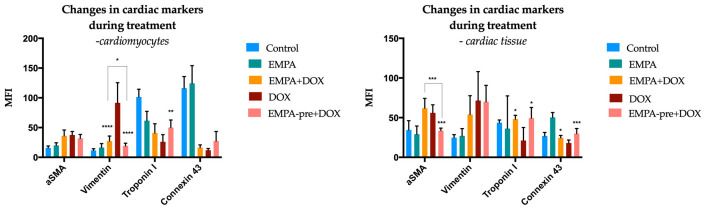
Treatment-associated changes in cardiac marker fluorescence intensity in isolated cardiomyocytes and cardiac tissue sections. Fluorescence microscopy images were analyzed to quantify the mean fluorescence intensity (MFI), calculated as the integrated density per unit area, for αSMA, vimentin, troponin I (cTnI), and connexin 43 (Cx43). Data are presented as mean ± SD, *n* = 8 per group. Each marker was analyzed independently by one-way ANOVA followed by Dunnett’s multiple comparisons test, using DOX as the reference condition. Dunnett-adjusted comparisons were used to determine whether EMPA + DOX or EMPA-pre + DOX differed from DOX. The direct comparison between EMPA + DOX and EMPA-pre + DOX was evaluated as a preplanned secondary comparison using an unpaired Welch’s *t* test. Statistical significance is indicated on the graph; * *p* < 0.05, ** *p* < 0.01, *** *p* < 0.001, **** *p* < 0.0001.

DOX treatment altered the fluorescence intensity profile of cardiac injury/remodeling markers compared with the control condition. EMPA administration, either simultaneously with DOX or as pretreatment before DOX exposure, generally shifted marker expression, consistent with a partial rescue of the DOX-induced phenotype. This effect reached statistical significance for selected markers when EMPA + DOX or EMPA-pre + DOX were compared with DOX using Dunnett’s multiple comparisons test. Direct comparison of the two EMPA regimens showed significant differences only for vimentin in isolated cardiomyocytes and αSMA in cardiac tissue sections, indicating that pretreatment was not consistently superior to concomitant EMPA treatment across all markers (Figure 15).

## 4. Discussion

Anthracycline-induced cardiotoxicity (AIC) is still a major challenge in cancer treatment, and researchers are showing increased interest in using machine learning (ML) to improve the prediction and detection of treatment-related cardiac dysfunction.

To the best of our knowledge, this preclinical study is the first to employ an unsupervised machine learning (ML) approach to derive a composite point cloud cardiotoxicity score that quantitatively captures LV mechanical dysfunction in vivo. The beating heart was visualized with a stereoscopic camera after thoracotomy in intubated, mechanically ventilated rats that received different doxorubicin (DOX) and empagliflozin (EMPA) treatment schedules. Rather than relying on invasive sensors that can alter local hemodynamics, we tracked dynamic structural deformation of the epicardial surface. By evaluating the generated 3D point cloud frame-to-frame, we extracted five spatial metrics: cluster count, noise ratio, in-cluster size, intra-cluster compactness, and fragmentation index. To capture the dynamic kinematic profile of the beating heart, the temporal mean and standard deviation of these variables were calculated across the video sequences.

A fundamental aspect of our methodology was the internal cross-validation of the unsupervised point cloud cardiotoxicity score (stratified on a 1.0-to-10.0 scale) by directly correlating it with a composite electrocardiographic (ECG) classification index derived from the same dataset. This robust index was designed to integrate critical dimensions of in vivo electrophysiological function, including QRS and PR interval durations, the QT interval, R- and T-wave amplitudes, and heart rate. These parameters, detailed in our multiparametric correlation analysis, serve as the biological reference for the kinematic score derived from 3D point cloud tracking in this preclinical setting. Statistical evaluation utilizing both Pearson and Spearman correlation coefficients revealed a highly significant association between the composite ECG index and the point cloud score. While the Pearson correlation (Pearson’s r = 0.954, *p* < 0.001) demonstrates a clear linear relationship between structural fragmentation and ECG parameters (most notably evidenced by the QT interval and QRS duration), the Spearman coefficient (ρ = 0.924, *p* < 0.0001) confirms the monotonic consistency of these alterations across all grades of induced cardiotoxicity. The rigorous cross-validation between specific spatial-kinematic metrics and the composite ECG index demonstrates that our automated scoring system reflects electromechanical uncoupling. Doxorubicin-induced structural degradation (e.g., myofibrillar loss, fibrosis, and cardiomyocyte atrophy) leads to a loss of coordinated epicardial wall motion. The point cloud captures this as increased spatial fragmentation. This mechanical descriptor is complementary to, rather than a replacement for, time-domain ECG intervals. It assesses the structural and mechanical consequences of injury, whereas the ECG reflects the electrical effects. Both measures converge on the same underlying pathological process, as demonstrated by their strong correlation. Ultimately, this precise alignment between computer vision metrics and classical electrophysiological markers confirms that EMPA treatment modulates the macroscopic architecture of myocardial contraction and safeguards the integrity of the cardiac conduction system against DOX-induced cardiac injury.

Although ML has been increasingly applied in cardio-oncology, most existing studies differ substantially from the present work in both their objectives and data structures. These studies predominantly use ML to predict the risk of cardiotoxicity in patients by integrating clinical, imaging, ECG, and biomarker features. For example, Chen et al. developed an XGBoost-based model that combined demographic, clinical, echocardiographic, ECG, and biomarker data to predict cardiovascular complications in breast cancer patients undergoing chemotherapy. Their model performed well and demonstrated the benefits of using multiple data types for risk assessment in cardio-oncology [36]. Similarly, Ahmadi et al. used radiomics and interpretable ML on 2D echocardiographic images taken before and after chemotherapy. They found that texture and shape features from standard views could predict cardiotoxicity with high accuracy, as confirmed by PermFIT and SHAP analyses for feature importance [37]. Chaix et al. combined clinical risk factors with genetic information in childhood cancer survivors, supporting the development of a predictive model for late AIC, and highlighting novel autophagy-related gene targets for future cardioprotective drug development [38]. Yagi et al. developed and validated an artificial intelligence (AI) model (AI-CTRCD) to predict CTRCD from a baseline 12-lead ECG obtained before anthracycline treatment. A high AI-CTRCD score was linked to a greater risk of CTRCD (adjusted hazard ratio 2.57; 95% CI 1.62–4.10), even after accounting for baseline LVEF, cancer type, other health conditions, and visible ECG changes. When the AI-CTRCD score was added to clinical predictors, the time-dependent area under the curve (AUC) at 2 years improved compared to using clinical factors alone (0.78 vs. 0.74). Simulations showed that using the AI-CTRCD score to select patients for follow-up echocardiograms could be a useful tool to improve cardio-oncology screening, especially when resources are limited [39]. Recent reviews point out that most AI applications now focus on risk stratification, diagnosis, and prognosis. They also highlight the need for more diverse methods and stronger connections between imaging and mechanistic data [40,41]. Unlike these approaches, our study uses machine learning to analyze in vivo left ventricular motion in a DOX-treated rat model and to derive a cohort-relative composite score of DOX-induced mechanical impairment in this preclinical setting.

Beyond clinical applications, preclinical studies are now looking at how ML can help detect AIC early in animal models. One recent study published by Mohammed et al. used ML to classify ECG signals and improve early detection of DOX-induced cardiotoxicity. Results showed that supervised algorithms can spot dose-related ECG changes more accurately than traditional interval analysis. Their findings suggest that using AI with high-resolution ECG data can improve the detection of subtle electrical toxicity in vivo [42].

The cellular composition of the heart includes both permanent and transient cell populations. Permanent cardiac cells include not only cardiomyocytes, which are numerically dominant, but also cardiac fibroblasts, endothelial cells, vascular smooth muscle cells, and other transient cell populations [43]. Where cardiomyocytes are primarily responsible for electrical impulse generation and contractile function, noncardiomyocyte populations are important for extracellular matrix homeostasis, angiogenesis, inflammatory signaling, and autonomic regulation [44]. Although the cardiotoxic effects of anticancer therapies have been predominantly studied at the level of cardiomyocytes, with a major focus on oxidative stress and topoisomerase 2β–mediated DNA damage, these mechanisms alone do not fully account for the complexity of AIC [45]. Emerging evidence indicates that cardiotoxicity is modulated by the cardiac microenvironment, underscoring the need to interrogate the role of non-cardiomyocytes in AIC [46].

The present study further evaluated cellular and tissue-level alterations to investigate the cardioprotective effects of SGLT2 inhibition in the setting of DOX exposure. Rat heart cells were isolated and cultured, followed by immunofluorescence (IF) staining for cTnI, vimentin, α-SMA, and Cx43. Groups III (EMPA + DOX) and V (EMPA-pre + DOX) suggested improved sarcomere structure, reduced fibroblast and myofibroblast activation, and more typical gap-junction organization compared to group IV (DOX). IF staining of paraffin-embedded heart tissue using the same markers corroborated these findings. As far as we know, no previous rat study has combined DOX exposure, SGLT2 inhibitor treatment, and an immunofluorescence panel targeting cTnI, vimentin, α-SMA, and Cx43 to examine cardiomyocyte injury, stromal activation, and gap-junction changes in a single experiment.

Several recent studies have revealed the importance of cellular and tissue characterization of AIC [26,47,48]. Histology from our previous study showed that DOX induced acute myocardial injury, including cytoplasmic vacuolization, loss of transverse striations, diffuse inflammation, thinning and elongation of cardiomyocytes, and cardiomyocyte atrophy. These changes were markedly attenuated in rats who received EMPA concomitantly or as a preconditioning treatment. In the chronic response pattern, DOX monotherapy showed extensive interstitial collagen deposition, whereas EMPA co-treatment showed less myocardial fibrosis, suggesting mitigation of structural remodeling [27]. Lee et al. recently reported that DOX exposure increases cardiac expression of α-SMA and vimentin by Western blot but did not detect differences in fibroblast populations by immunohistochemistry, implying that these markers also reflect smooth muscle, endothelial, and stromal cell activation rather than fibroblasts alone [49].

The novelty of the present work lies at the intersection of advanced cardiac kinetic analysis using AI, preclinical modeling, and SGLT2-mediated cardioprotection in cardio-oncology. Unlike most clinical studies that focus on global longitudinal strain (GLS), we apply Fourier-domain and point-cloud-based characterization of LV mechanics in a rat DOX model, addressing a largely unexplored dimension of myocardial deformation in this field. In contrast to existing preclinical AI applications, which typically focus on ECG classification or omics, our study is, to our knowledge, the first to combine ML with high-resolution kinetic imaging in Sprague–Dawley rats exposed to anthracyclines. Furthermore, by evaluating EMPA’s effects on both conventional (ECG) and ML-derived kinetic signatures, alongside immunofluorescence markers, we provide an integrated mechanistic framework that extends beyond current data on SGLT2 inhibitors in cardio-oncology. Together, these parts help bridge the gap between experimental cardiotoxicity models and AI risk prediction efforts.

A limitation of this study is that the correlations provide supportive, internal cross-validation within a small preclinical dataset (*n* = 18) and should be interpreted as exploratory, despite their strength, given the small sample size. External validation in larger cohorts is required before any inference about generalizability can be made. The model is preclinical and relies on open-chest, mechanically ventilated rats with direct visualization of the beating heart, serving as an experimental phenotyping platform rather than a near-term clinical surveillance tool. Its purpose is to provide objective, high-fidelity mechanical readouts in experimental cardio-oncology. It should also be emphasized that the point cloud score quantifies myocardial mechanical kinetics, whereas the ECG served as an internal electrophysiological reference for cross-validation. Given the well-known interspecies differences in cardiac electrophysiology—most notably the absence of a prominent plateau phase in the rodent action potential and the substantially higher HR—the present pipeline is neither intended for nor should be extrapolated to human ECG datasets. Clinical translation would require fundamentally different, non-invasive acquisition and dedicated human validation. These limitations do not negate the proof-of-concept value of the method but delineate the scope within which the results should be interpreted.

The available data do not support the hypothesis that the point cloud score detects cardiotoxicity earlier than the ECG index. Establishing such temporal precedence would require repeated-measures acquisition, which we propose as a direction for future work.

## 5. Conclusions

This study provides a preclinical proof of concept that machine learning analysis of 3D point clouds of epicardial motion can phenotype anthracycline-induced cardiotoxicity and capture the cardioprotective signal of empagliflozin in this rat model. The algorithm consistently indicated that concurrent treatment with empagliflozin was associated with improved cardiac kinetics compared with doxorubicin monotherapy, consistent with the differences observed in the ECG index. These findings suggest that the method can detect the cardioprotection effect of SGLT2 inhibition and support the potential of ML-based stereoscopic imaging for preclinical cardio-oncology research.

Beyond functional assessment, the study incorporated a cellular and tissue-level perspective using a multimarker immunofluorescence panel to characterize cardiomyocyte injury, stromal activation, and gap-junction remodeling.

This multimodal approach illustrates the potential of combined computational and biological results to refine preclinical cardio-oncology models.

## Figures and Tables

**Table 1:** Depth value ranges and corresponding point cloud geometry (MiDaS inverse relative depth, 384 × 384 px frames).

Anatomical Region	Typical Depth Value	Point Cloud Appearance
Heart center—peak systole	0.483–0.495	Apex of dome—highest point
Heart center—diastole	0.498–0.528	Dome slightly reduced in height
Heart wall/slope	0.528–0.610	Sides of the dome
Surrounding tissue	0.610–0.680	Flat surrounding plain

## Data Availability

The original contributions presented in the study are included in the article. Further inquiries can be directed to the corresponding author.

## Abbreviations

The following abbreviations are used in this manuscript:

α-SMA	Alpha-smooth muscle actin
AIC	Anthracycline-induced cardiotoxicity
AU	Arbitrary units
BMP	Beats per minute
CK	Creatine kinase
cTnI	Cardiac troponin I
CTRCD	Cancer therapy-related cardiac dysfunction
Cx43	Connexin 43
DAPI	4′,6-diamidino-2-phenylindole
DOX	Doxorubicin
DPBS	Dulbecco’s phosphate-buffered saline
ECG	Electrocardiogram
EF	Ejection fraction
EMPA	Empagliflozin
FBS	Fetal bovine serum
FS	Fractional shortening
FSP	Frames per second
HF	Heart failure
HR	Heart rate
IF	Immunofluorescence
kNN	k-Nearest Neighbors
LV	Left ventricle
LVEF	Left ventricle ejection fraction
MFI	Mean fluorescence intensity
ML	Machine learning
MRI	Magnetic resonance imaging
NLRP3	Nucleotide-binding domain-like receptor protein 3
NT-proBNP	N-terminal pro-B-type natriuretic peptide
Pen Strep	Penicillin-Streptomycin
QTc	Corrected QT
R-Amp	R-wave amplitude
RF	Random Forest
ROIs	Regions of interest
ROS	Reactive oxygen species
SD	Standard deviation
SGLT2	Sodium-glucose cotransporter 2
STZ	Streptozotocin
SVM	Support Vector Machine
T-Amp	T-wave amplitude
